# Recent advances in MXene-based force sensors: a mini-review

**DOI:** 10.1039/d1ra02857j

**Published:** 2021-05-26

**Authors:** Dongchen Tan, Chengming Jiang, Xuguang Cao, Nan Sun, Qikun Li, Sheng Bi, Jinhui Song

**Affiliations:** Key Laboratory for Precision and Non-traditional Machining Technology of the Ministry of Education, Dalian University of Technology Dalian 116024 China jiangcm@dlut.edu.cn jhsong@dlut.edu.cn

## Abstract

As an emerging two-dimensional (2D) material, MXene has excellent conductivity and abundant surface functional groups. Its unique layered structure, large surface area, and prominent hydrophilicity show remarkable performances, which allow abundant possibilities to work as the sensing element alone or combined with other auxiliary materials. As a senior member of MXenes, Ti_3_C_2_T_*x*_ has shown great potential in the development of force sensors. The research development of force sensors based on Ti_3_C_2_T_*x*_ MXene is reviewed in this paper, presenting the advanced development of force sensors in various forms and summaring their current preparation strategies and characteristics. In addition, the corresponding challenges and prospects of the MXene-based sensors are also discussed for future research.

## Abstract 2

As an emerging two-dimensional (2D) material, MXene has excellent conductivity and abundant surface functional groups.

## Graphical abstract

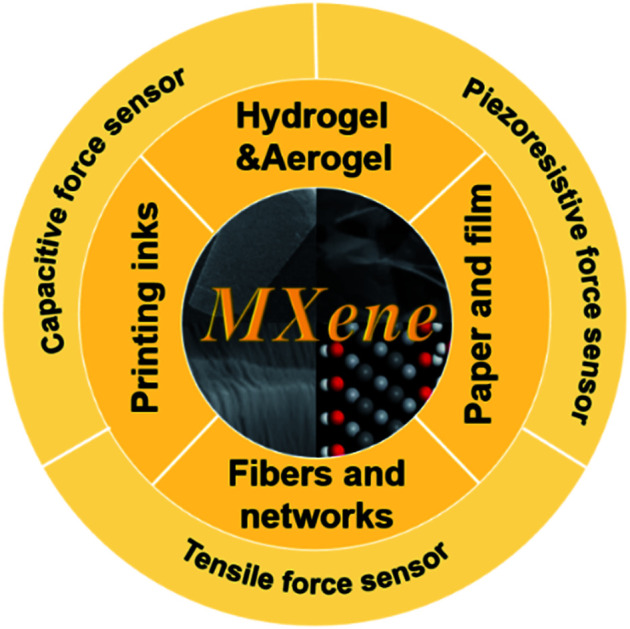

## 1. Introduction

As indispensable electronic devices, sensors have been widely used in wearable devices, environmental monitoring, electronic skin, artificial intelligence, human–computer interactions, and other fields.^1–6^ In order to achieve specific functions, bionic structures inspired by biology or mechanical two-dimensional structures (such as serpentine structures) and three-dimensional (3D) structures (such as island-bridge structures) that can achieve large stretching and bending properties are proposed. However, the selection of materials is more important than structure because the characteristics of the materials are the key to performance. So far, many materials have been proposed to fabricate high-performance sensors, one-dimensional (1D) materials and two-dimensional (2D) materials have become the focus of research. MXene stands for layered transition metal carbides or nitrides, consisting of more than 60 kinds, such as Ti_2_C, V_2_C, Nb_2_C, Ti_4_N_3_, TiNbC, Ti_3_CN, and Mo_2_TiC_2_.^7–9^ MXenes are obtained by chemical treatment from precursors of the M_*n*+1_AX_*n*_ phase (often referred to as the MAX phase), where M is an early transition metal, A is usually an IIIA or IVA element, X is C and/or N, and *n* = 1, 2, 3.^10^ A series of exciting properties make MXenes a hotspot of research, expecting to pave the way for future of sensors.

**Fig. 1:**
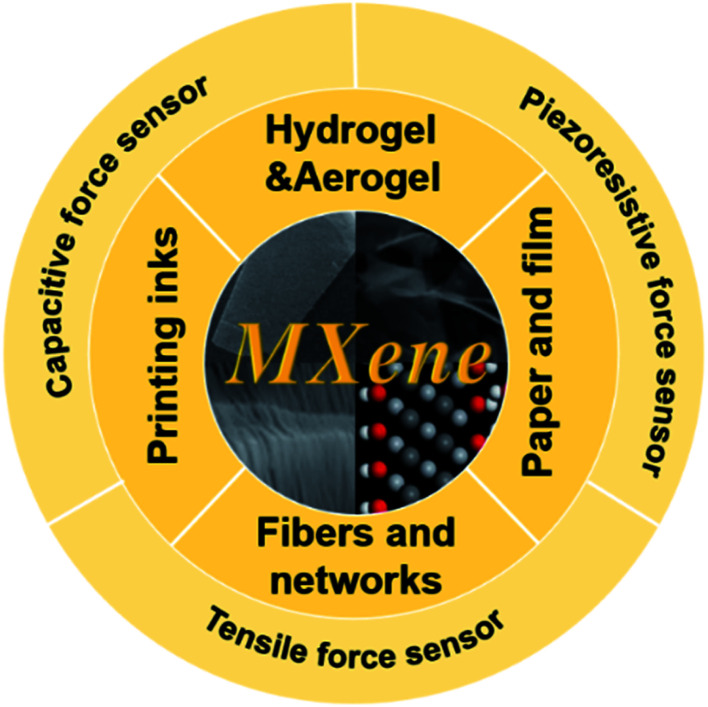
Review content, from four perspectives, including aerogel and hydrogel, fiber and network, ink and printing, film and paper, summarized MXene-based pressure resistance sensor, capacitive force sensor, and tensile force sensor.

This review focuses on the application of Ti_3_C_2_T_*x*_ MXene in force sensors, including piezoresistive force sensors, tensile force sensors, and capacitive force sensors. Its high conductivity, mechanical stiffness, and diverse presentation states make it an attractive choice for force sensors. The structure of the review is shown in Fig. 1. Piezoresistive force sensor, capacitive force sensor, and tensile force sensor are introduced respectively from four perspectives: aerogel and hydrogel, fiber and network, ink and print, film and paper. Firstly, we briefly summarized the preparation and characterization of Ti_3_C_2_T_*x*_ MXene. Then, the evaluation standard and working mechanism of the force sensors are summarized. Thirdly, the development of Ti_3_C_2_T_*x*_ MXene force sensors on structures and materials is reviewed. Last, we conclude with the discussion of the future development of sensors with Ti_3_C_2_T_*x*_ MXene.

## 2. Preparation and properties of MXene

### 2.1 The preparation of MXene

**Fig. 2:**
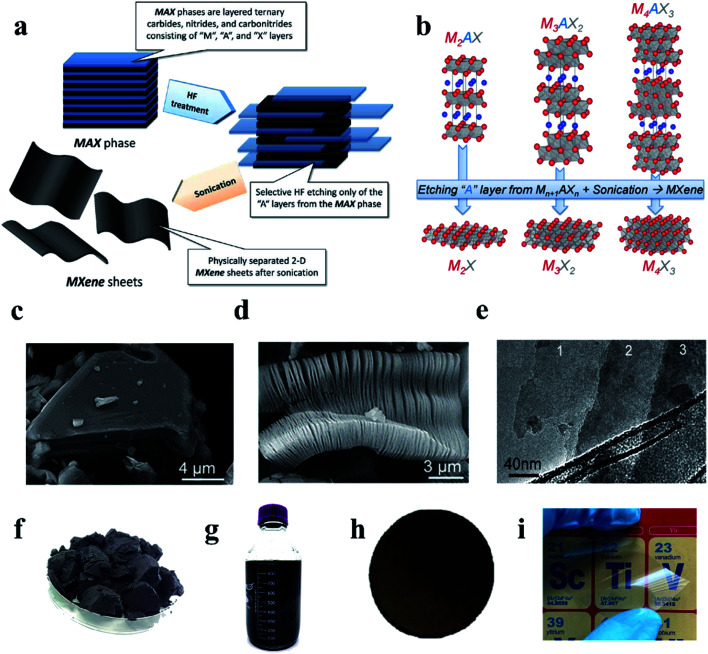
(a) Schematic diagram of typical reaction process for preparation of MXene by etching MAX phase with HF.^28^ (b) Schematic diagram of atomic structure changes during the reaction from MAX phase to MXene.^28^ (c) The SEM of the MAX phase (Ti_3_AlC_2_). (d) Organ-like Ti_3_C_2_T_*x*_ MXene.^28^ (e) A monolayer Ti_3_C_2_T_*x*_ MXene. (f) Freeze-dried powder of Ti_3_C_2_T_*x*_ MXene.^28^ (g) Ti_3_C_2_T_*x*_ MXene dispersion.^28^ (h) Ti_3_C_2_T_*x*_ MXene membrane was prepared by vacuum filtration.^28^ (i) Transparent film based on Ti_3_C_2_T_*x*_ MXene.^29^

In 2011, scientists at Drexel University prepared the first MXene by etching the MAX phase with hydrofluoric acid (HF).^11,12^ Subsequently, the family has sprung up in research and development, and its types and applications have been greatly expanded. Fig. 2a shows a schematic diagram of the preparation of MXene using HF etching of MAX phases. The structure of MAX phases and the corresponding MXenes are shown in Fig. 2b and Ti_3_C_2_T_*x*_ MXene is prepared from Ti_3_AlC_2_ phases (Fig. 2c). In traditional manufacturing methods, organ-like Ti_3_C_2_T_*x*_ MXene (Fig. 2d) is produced by HF etching, which allows the Ti_3_AlC_2_ phases to be layered well during etching, but often does not form a perfect monolayer of Ti_3_C_2_T_*x*_ MXene sheets. By improving the preparation scheme, lithium fluoride (LiF) and hydrochloric acid (HCl) are introduced to etch Ti_3_AlC_2_ phases and formed a large-scale monomer (Fig. 2e) under the action of ultrasound.^13^ In the reaction process of Ti_3_AlC_2_ and HF, the reaction mechanism is as follows:1
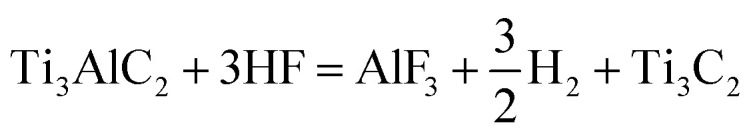

2 Ti_3_C_2_ + 2H_2_O = Ti_3_C_2_(OH)_2_ + H_2_

3 Ti_3_C_2_ + 2HF = Ti_3_C_2_F_2_ + H_2_

In addition, the molten salt method is also a feasible and effective route for the synthesis of MXene.^14–16^ Compared with the preparation of MXene using HF etching MAX phase, the molten salt method greatly reduces the safety risks and environmental pollution problems. Fluoride molten salt method^17^ and Lewis acid molten salt method^18,19^ are both effective strategies. By preparing MXenes with Lewis acid salt method, Kamysbayev *et al.* controlled the surface groups through substitution and elimination reactions. MXene with O, NH, S, Cl, Se, Br, Te surface terminal and MXene without surface terminal have been successfully synthesized.^20^ To obtain better quality monolayer MXene, many new improvements have been proposed. *N*,*N*-Dimethylformamide (DMF),^21,22^*N*-methyl-2-pyrrolidone (NMP),^23^ and DMSO^24–26^ are proved to be “good solvents” for Ti_3_C_2_T_*x*_ flakes by Gogotsi *et al.*^27^ Zhang *et al.* successfully developed a tuned microenvironment method (TMM) for the preparation of highly concentrated MXene organic solvents to obtain a large delaminated product yield, which can easily achieve a yield of 63.9% for a single layer of MXene and close to 100% if the material is continuously collected.^21^ Better solubility and single-layer MXene production will significantly increase productivity and reduce costs, laying a good foundation for large-scale hardware fabrication.

MXene can be presented in many forms, such as lyophilized powder formed by freeze-drying MXene solution (Fig. 2f),^28^ suspension formed by ultrasonic centrifugation (Fig. 2g),^28^ thick film MXene formed by suction filtration (Fig. 2h),^28^ the single-layer transparent film formed by deposition (Fig. 2i),^29^*etc.* The variety of presentations also foreshadows the variety of sensor manufacturing.

### 2.2 Characterization and properties of MXene

Compared with other functional materials, MXenes are characterized by their electrical conductivity, large specific surface area, unique layered structure, satisfactory dispersity and abundant dispersity terminal group in aqueous solution opens a new door for sensor research and development.^30–32^ As presented in Table 1, in contrast, Ti_3_C_2_T_*x*_ MXene not only has high conductivity but also has a high surface area and abundant surface functional groups.^28^

**Table 1:** Comparison of basic properties of the MXene family and other materials

Type	Electrical conductivity [S m^−1^]	Surface areas	Terminal groups
Ag bulk	6.3 × 10^7^	Low	—
Cu bulk	5.96 × 10^7^	Low	—
Au bulk	4.10 × 10^7^	Low	—
Carbon nanotube	∼10^6^	High	–COOH
Graphene	∼10^8^	High	–COOH
reduced graphene oxide	1.6 × 10^5^	High	–COOH
Ti_3_C_2_T_*x*_ film^4^	2.4 × 10^6^	High	–F, –OH, and –O
Ti_3_C_2_T_*x*_ flakes^41^	4.6 × 10^6^	High	–F, –OH, and –O
Epoxy/Ti_3_C_2_T_*x*_^102^	4.52 × 10^−4^	High	–F, –OH, and –O
Ti_3_C_2_T_*x*_ film (after annealing treatment)^103^	1.51 × 10^−7^	High	–F, –OH, and –O

#### 2.2.1 Environmental stability

Ti_3_C_2_T_*x*_ MXene is easy to be oxidized in the air and water, so its environmental stability is the key research content, which is related to the stability and service life in the process of sensor preparation and use. Oxidation starts at the edge of MXene and then spreads to the whole MXene.^33^ Oxidation occurs in almost all common media and the rate of oxidation is the fastest in the liquid form and slows down in the solid form. The oxidation process can be described as

4 Ti_3_C_2_O_2_ + 4H_2_O = 3TiO_2_ + 2C + 4H_2_

**Fig. 3:**
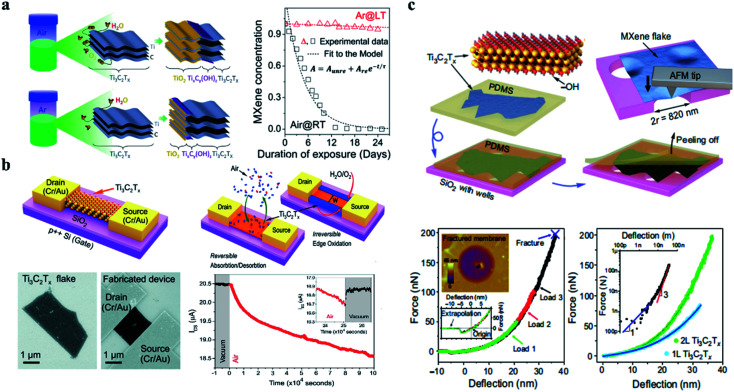
(a) Oxidation process and rate of Ti_3_C_2_T_*x*_ MXene dispersion in air and argon.^34^ (b) The schematic diagram of an electrical performance measurement device based on Ti_3_C_2_T_*x*_ MXene.^41^ (c) The measurement method and process diagram of Young's modulus of Ti_3_C_2_T_*x*_ MXene.^44^

By studying the oxidation rates of Ti_3_C_2_T_*x*_ MXene solutions in different environments, as presented in Fig. 3a, Zhang *et al.* concluded that a reliable and practical storage method was to store Ti_3_C_2_T_*x*_ MXene colloidal solution in a sealed argon gas (Ar) bottle at 5 °C. In addition to Ti_3_C_2_T_*x*_ MXene solutions, great attention should be paid to the oxidation during the use of freeze-drying or vacuum drying.^34^ In general, one of the difficulties faced by MXene in the process of use is its fragile oxidation resistance, and it is worth exploring how to solve the performance loss caused by oxidation. Ti_3_C_2_T_*x*_ MXene is readily converted to TiO_2_, and in the study of Naguib's team and Li *et al.* Ti_3_C_2_T_*x*_ is completely converted by annealing at 1150 °C for the 30 s or annealing at 400 °C.^29,35^ This oxidation process often sacrifices some advantages of MXene, such as the reduction of conductivity and the loss of capacitance. It is worth mentioning that the ionic group of polyphenylene can effectively occupy the edge of the MXene sheet, thus preventing MXene from being oxidized by water molecules.^36^ High-temperature annealing^37^ and silicification in hydrogen^38^ can improve the oxidation resistance of MXene to a certain extent. How to avoid the loss of stability caused by oxidation is of great significance for practical applications and device fabrication, because in the process of material synthesis, storage, device manufacturing, and use will inevitably undergo high temperature or oxidation process.^39^

#### 2.2.2 Electrical properties

For a single layer of Ti_3_C_2_T_*x*_ MXene, the electrical properties are worth investigating, to explore its electrical performance in a deeper level to show its performance characteristics, in the application of micro and nanosensors to lay a foundation. According to the calculation of density function theory, the results show that the electronic structure of Ti_3_C_2_T_*x*_ is heavily dependent on –F and –OH. Their presence makes the metal Ti_3_C_2_ transform into a semiconductor with a bandgap of 0.05 eV and 0.1 eV, respectively, which reduces the carrier density and thus reduces the conductivity.^40^ Lipatov *et al.* demonstrated a method to measure the conductivity of a single layer of MXene and the effect of oxidation in the environment on the conductivity of the MXene. Fig. 3b shows the schematic diagram of an electrical performance measurement device based on Ti_3_C_2_T_*x*_. On a Si/SiO_2_ substrate, Ti_3_C_2_T_*x*_ plates are bridged with a Cr/Au source and drain electrodes, and a back-gate electrode, a highly P-doped conductive silicon, through a SiO_2_ medium of 300 nm. The sample is provided with a drain–source voltage at room temperature and was vacuumed for two days before that to reduce the influence of surface adsorbents. The average resistivity was 2310 ± 570 Ω aq^−1^. Similarly, the change of electrical properties of Ti_3_C_2_T_*x*_ in the air with the oxidation process is also studied.^41^

#### 2.2.3 Mechanical properties

In addition to oxidation and electrical properties, the mechanical properties of Ti_3_C_2_T_*x*_ are also the focus of attention.^42^ The theoretical calculation of Young's modulus of Ti_3_C_2_T_*x*_ can reach 502 GPa.^43^ Lipatov *et al.* also studied the young's modulus of Ti_3_C_2_T_*x*_. As shown in Fig. 3c, the monolayers and bilayers Ti_3_C_2_T_*x*_ are transferred to SiO_2_ substrate with special holes. The nanoindentation is carried out at the tip of the atomic force microscope and the force–displacement curve is recorded. The calculated results show that the effective Young's modulus of monolayer Ti_3_C_2_T_*x*_ is 0.33 ± 0.03 TPa.^44^ The nanoindentation experiment results show that the nanoindentation properties of Ti_3_C_2_T_*x*_ are higher than that of graphene oxide (GO), reduced graphene oxide (RGO), and MoS_2_, but lower than that of hexagonal boron nitride (h-BN) and graphene. In addition, Ti_3_C_2_T_*x*_ MXene-based films and papers also exhibit good macro mechanical stability, maintaining macro stability during bending and stretching.^45^

The research on the performance of single-layer Ti_3_C_2_T_*x*_ MXene has revealed its basic properties and laid a foundation for the development and application of subsequent microsensors, although the research on the microsensors based on a single layer or multilayer Ti_3_C_2_T_*x*_ MXene is still insufficient.

## 3. Evaluation criteria and working mechanism

### 3.1 Performance evaluation standard of MXene force sensors

Sensitivity, sensing range, and response time are the core performance of the force sensor, which determines the minimum induction limit, sensing speed and maximum strength bearing limit of the sensor to detect external strain.^6,46–49^ Sensitivity, also known as gauge factor (GF), is expressed by the slope of the relative change of the resistance or capacitance signal to the external strain of the sensor during its operation.^33^ For resistive sensors, GF is denoted as5
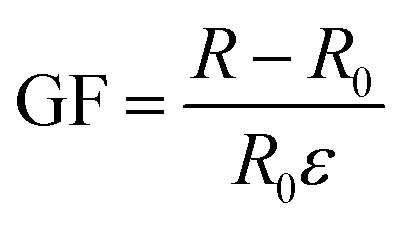


For capacitive sensors, GF is denoted as6
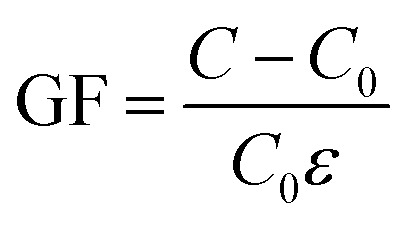
where *ε* is the strain, *R*_0_ and *C*_0_ are respectively the resistance and capacitance without external action, *R* and *C* are respectively the resistance and capacitance after tensile deformation under external force.

The sensitivity of the force sensor is expressed as7
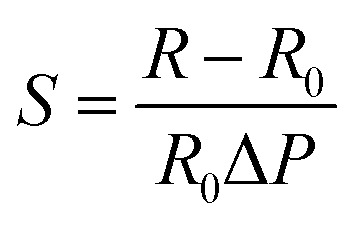
where *R*_0_ is the resistance when no load is applied, *R* is the resistance when the load is applied, and Δ*P* is the relative change of the pressure load.

The sensing range is the working window of the sensor, that is, the minimum and maximum power range that can work. Response time indicates the response speed of the sensor to the force signal input from the external environment. In addition, service life, linearity, hysteresis, and creep properties are the criteria for evaluating force sensors, and efforts should be made to optimize these properties while maximizing sensitivity, induction range, and response time.^50–52^

The practicability of sensors is also a complex but powerful evaluation criterion. Appropriate volume, sufficient stability, and a wide working range are the criteria we need to consider. For implantable devices, biocompatibility, long-time performance, and the discrimination of internal noise need to be considered. For wearable sensors, comfort, aesthetics, working stability and other conditions need to be considered comprehensively. For electronic skin, in the face of strong external force, it has buffer protection, the perception of force spatial distribution, environmental stability is the primary consideration. This is a complex evaluation system, and there are no perfect and authoritative evaluation criteria.

### 3.2 Working mechanism of force sensors based on MXene

#### 3.2.1 Piezoresistive force sensor

Piezoresistive force sensor based on MXene realizes the sensing function of force signal by using the change of the resistance under the action of force. The unique layered structure of the organ-like MXene and its diverse intercom position with other materials, such as hydrogels, aerogels, inks, films, and paper, provide the basis for the development of piezoresistive force transducers based on MXene.

**Fig. 4:**
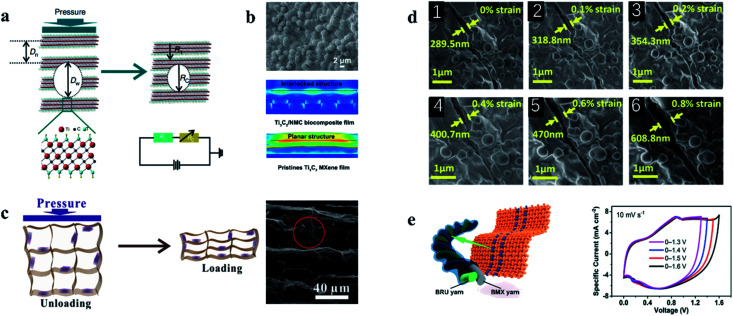
(a) Schematic diagram of piezoresistive effect mechanism of organ-like MXene.^53^ (b) Biologically inspired piezoresistive sensor based on MXene and its working principle finite element simulation analysis.^54^ (c) The pore structure of the aerogel based on MXene changes the resistance during the stress process.^55^ (d) Resistance changes induced by tensile cracks in MXene-based sensors.^57^ (e) Structure of capacitive sensor based on MXene.^58^

As shown in Fig. 4a, the laminated structure of organ-like MXene is very favorable for the preparation of piezoresistive sensors. During the process of force, the distance between the laminated MXene changes, causing the resistance to change.^53^ Moreover, the conversion of force signal to resistance value is realized by structural design. This kind of structure often uses force deformation to realize the design of the piezoresistive sensor by changing the contact area. Fig. 4b shows a piezoresistive sensor design that takes advantage of structural deformations resulting in changes in resistance values, and its finite element simulation analysis shows the working principle of stress deformations.^54^ Hydrogels and aerogels are the main forces in the design of piezoresistive sensors. The specific pore structure of aerogels is conducive to the generation of piezoresistive effect. Fig. 4c shows the working principle of porous aerogels.^55^

#### 3.2.2 Tensile force sensor

In general, the tension sensor takes advantage of the resistance change caused by the deformation of the microstructure of the MXene-based material under the action of tension during the tensile process. Hydrogels are a very promising option, and their tensile properties of more than 3400% in previous work provide a good basis for the preparation of tension sensors.^56^ During the tensile strain process, the hydrogels changed the gap between the MXene nanosheets that were connected face to face and changed the surface contact between the MXene nanosheets, which caused the resistance to be changed.

Another form of tensile force sensor takes advantage of the change in resistance values caused by the change in the spacing of material cracks. Fig. 4d shows the scanning electron microscopy (SEM) images of MXene films under different strain states. As the strain increases, the crack edges separate, resulting in an increase in resistance.^57^

#### 3.2.3 Capacitive force sensor

The capacitive sensor is a kind of sensor that takes the capacitor as the sensing element in the process of acceptance and converts the deformation into the capacitance change.^1^ As a conductive material, MXene has great potential in the development of capacitive sensors. As shown in Fig. 4e, the prototype diagram of energy textiles based on MXene and the working condition of capacitive sensors based on the textiles are shown.^58^

## 4. Force sensors based on Ti_3_C_2_T_*x*_ MXene

The force sensors are widely used and have been extensively studied by researchers. They are especially important in the field of force perception and force response and are widely used in environmental monitoring, intelligent robot, human–computer interaction, medical detection, motion monitoring, electronic skin, and so on. The advantages of MXene, including its electrical conductivity, flexibility, and hydrophilicity, make it a potential application in force sensors. In this section, we review the latest developments in force sensors, including force-induced strain sensors and force-induced compression sensors.

### 4.1 MXene based hydrogel/aerogel for force sensors

Thin-film sensors usually show a low sensitivity due to the limited compression space. Conductive hydrogels,^59–62^ aerogels^63–66^ with larger deformation space, and excellent mechanical performance including bending resistance, tensile resistance, and torsion resistance are widely used in the piezoresistive pressure sensor, not only such, the specially designed hydrogels and aerogels have self-healing properties and environmental stability, which can also remain stable in the face of extremely high or low-temperature environments, providing a powerful boost for the application of sensors.

**Fig. 5:**
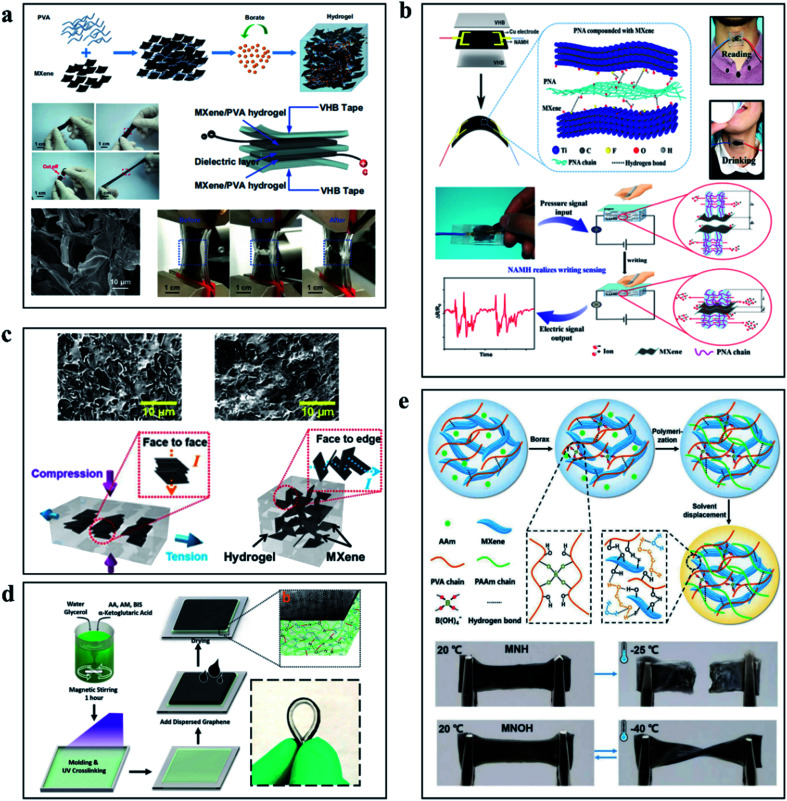
(a) The hydrogel based on PVA/Ti_3_C_2_T_*x*_ MXene, including a schematic of the preparation process, tensile display and sensor structure.^67^ (b) Schematic diagram of hydrogel preparation process based on PNA and Ti_3_C_2_T_*x*_ MXene and the working diagram of the force sensor based on PNA/Ti_3_C_2_T_*x*_ MXene hydrogel.^68^ (c) Schematic diagram of internal changes of high-performance hydrogels based on Ti_3_C_2_T_*x*_ MXene during the tension process.^56^ (d) Schematic diagram and physical demonstration of preparation of hydrogel based on graphene and Ti_3_C_2_T_*x*_ MXene.^69^ (e) Preparation process and performance demonstration of low temperature resistant self-healing hydrogels.^70^

Force sensors, as an important component of electronic skin, aim to reproduce the information perception function of biological skin, with scalability and self-repair becoming the goals to be sought. As presented in Fig. 5a, Zhang and his team prepared a hydrogel with Ti_3_C_2_T_*x*_ MXene and polyvinyl alcohol (PVA), which has tensile and self-healing properties, as well as a capacitive strain sensor electronic skin.^67^ The addition of Ti_3_C_2_T_*x*_ MXene improved the conductivity of PVA and the self-healing property of hydrogel. The electrode is capable of withstanding up to ∼1200% tension and fully self-healing in ∼0.15 s. The linearity of the capacitive sensor based on this electrode is up to ∼200%, low hysteresis, favorable sensitivity (∼0.40), and maintained performance after 10 000 cycles. Relative to the self-healing ability, the pursuit of tensile capacity limits, in a wearable device to realize the weak signal detection, such as the force signal produced by joints bend, swallowing and pulse has become a research focus in the wearable sensors. Chen *et al.* developed a Ti_3_C_2_T_*x*_ MXene composite conductive hydrogel with tensile strain exceeding 1800%, which can detect weak force signals generated by the human body as a wearable sensor.^68^Fig. 5b shows the composition and sensing mechanism of the hydrogel. The potential of Ti_3_C_2_T_*x*_-based hydrogels is enormous, and the improvement of tensile strength and sensitivity is promising. The composite hydrogels prepared by Zhang *et al.* have a GF of 25, which can achieve more than 3400% tensile deformation, instantaneous self-healing ability.^56^Fig. 5c shows the deformation mechanism of this composite hydrogel. The affinity between Ti_3_C_2_T_*x*_ MXene and other materials provides the conditions for the preparation of the composite materials. The Lv team attempted to prepare the graphene/Ti_3_C_2_T_*x*_ composite hydrogel by pouring graphene solution on the hydrogel with glycerin as the co-solvent during the formation of the hydrogel substrate, as shown in Fig. 5d. The strong hydrogen bond formed between glycerol and water enables the hydrogel strain sensor to effectively retain water in the polymer network, which enables the hydrogel to obtain good temperature stability and improve the tensile properties of the hydrogel.^69^ Stability in extreme environments is urgently needed for certain sensors used in specific applications. By immersing ethylene glycol nanocomposite hydrogel (MNH) in ethylene glycol (EG) solution, Liao and colleagues developed an antifreeze, self-healing, and conductive polydiene nanocomposite organic hydrogel that can resist low temperatures of −40 °C and achieve a GF of 44.85.^70^ The work of Liu and colleagues is presented in Fig. 5e. These hydrogels have demonstrated strong mechanical and sensing properties for force sensors, especially for wearable devices and force sensors for electronic skin. But hydrogels still need to be explored on the way of volume miniaturization, function diversification, and integration.

**Fig. 6:**
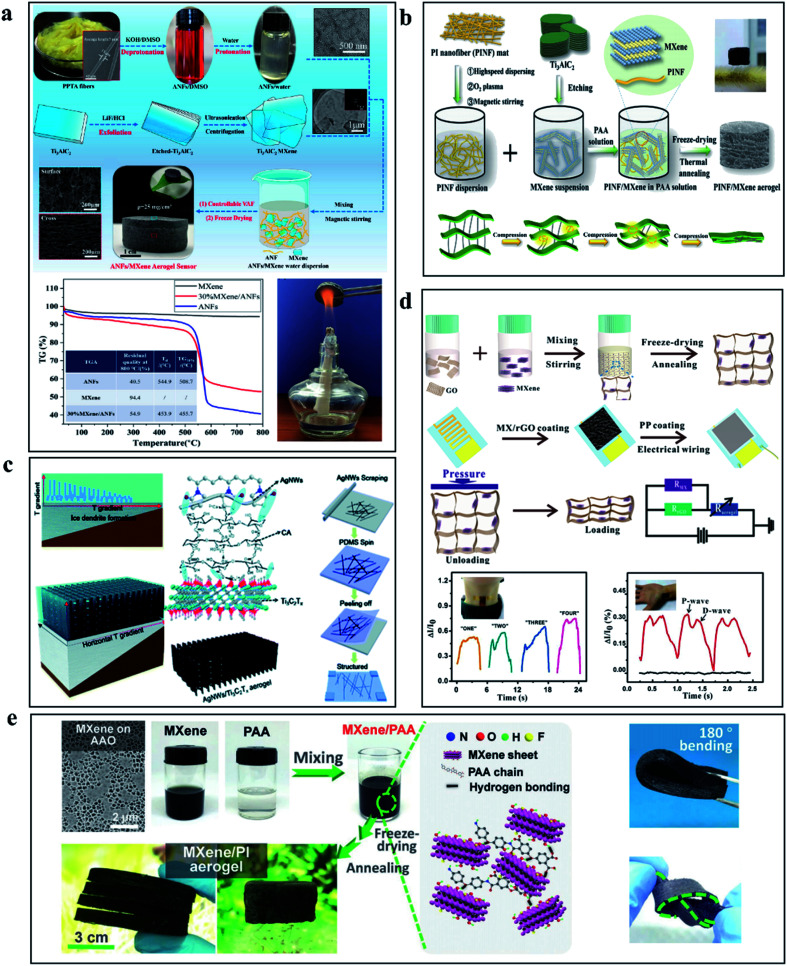
(a) Schematic diagram of preparation process and thermal stability of aerogel based on PPTA fiber and Ti_3_C_2_T_*x*_ MXene.^72^ (b) Schematic diagram of aerogel preparation process based on PI fiber and Ti_3_C_2_T_*x*_ MXene.^73^ (c) Schematic illustration of the preparation of Ti_3_C_2_T_*x*_ MXene/AgNWs aerogels using the directional freezing technique.^74^ (d) Fabrication diagram and performance characterization of force sensor based on graphite oxide (GO) and Ti_3_C_2_T_*x*_ MXene.^55^ (e) Preparation diagram and performance demonstration of aerogel based on PI and Ti_3_C_2_T_*x*_ MXene.^75^

Compared to the tensile properties of hydrogels, the porous structure of lightweight aerogels gives them excellent compressibility. The change of porosity during compression results in a wide range of resistance changes, which opens up many possibilities for the development of piezoresistive force sensors.^71^ The existing applications of piezoresistive sensors based on MXene are mainly based on the process of the change of the overall resistance of the material caused by the cracks or the reduction of layer spacing caused by the phenomenon of the stress process of MXene-based materials. Wang *et al.* creatively integrated thermally stable and fire-resistant acrylamide nanofibers (ANF) with MXene sheets and prepared composite aerogels through vacuum filtration. Instability of the 3D layering and “mortar–brick” porous structure makes the density is only 25 mg cm^−3^, the force sensor based on Ti_3_C_2_T_*x*_ MXene/ANF aerogel has a strain range of 2.0–80.0%, a sensitivity of 128 kPa^−1^, and can detect ultra-low force signals of 100 Pa. The addition of ANF also enables the aerogel sensor to demonstrate excellent flame retardancy and withstand extreme temperatures of 200 °C, the preparation process is shown in Fig. 6a.^72^ Intriguingly, as the work is shown in Fig. 6b, Liu and colleagues assembled a multilayered aerogel with Ti_3_C_2_T_*x*_ MXene using flexible PINF nanofibers. The porous structure and the strong bond between PINF and Ti_3_C_2_T_*x*_ MXene allow the PINF/Ti_3_C_2_T_*x*_ composite aerogel to exhibit ultra-low density with 9.98 mg cm^−3^ and withstand temperature variations from −50 to 250 °C, recover from up to 90% strain and remain stable after 1000 cycles of repeated compression. In addition, the composite aerogels also have superior oil–water separation capacity, high adsorption capacity (55.85–135.29 g g^−1^), and stable recoverability due to their hydrophobicity and solid layered porous structure. In the application of the force sensor, the PINF/Ti_3_C_2_T_*x*_ composite aerogel shows a super performance.^73^ From the preparation method presented in Fig. 6c, Bi *et al.* prepared AgNWs/Ti_3_C_2_T_*x*_ based aerogel by directional freezing strategy. Using the temperature gradient generated by the wedge-shaped device and the synergistic effect of 1D AgNWs and 2D Ti_3_C_2_T_*x*_, the sandwich method was combined with the conductive electrode of AgNWs/PDMS to construct the pressure sensor, and the 3D layered structure was obtained. The sensitivity of the aerogel sensor can reach 645.69 kPa^−1^ and the detection limit is 1.25 Pa. The performance remains stable after more than 10 000 compression cycles under 20% strain.^74^ The 3D porous structure is the focus of aerogels, without the use of polymer doping and special treatment methods, Ma *et al.* produced a hybrid 3D structure of MXene/RGO, which was obtained by freeze-drying. As presented in Fig. 6d, the piezoresistive sensor based on the hydrogel has a high sensitivity of 22.56 kPa^−1^ and can maintain satisfactory stability after 10 000 cycles, which has opened up a way to fabricate a high-performance aerogel sensor by a simple process.^55^ Because of the weak interaction between layers in MXene, it is difficult to build an independent flexible 3D framework. In Fig. 6e, Liu proposed an interfacial enhancement strategy using polyimide macromolecules to bridge the interaction of individual Ti_3_C_2_T_*x*_ MXene sheets to build a multifunctional, hyperelastic, and lightweight 3D Ti_3_C_2_T_*x*_ architecture. The light aerogels prepared by this method have high super elastic reversible compressibility, benign fatigue resistance, and high electrical conductivity (∼4.0 S m^−1^).^75^ As a kind of force sensor material with large compression and considerable piezoresistive properties, aerogels can give full play to the conductive advantages of Ti_3_C_2_T_*x*_ MXene and introduce special properties such as high-temperature resistance, so they have a broad application prospect. At the same time, we need to continue to think and explore more practical aerogel synthesis schemes.

### 4.2 MXene based fibers and networks for force sensors

Fiber and network manufacturing technologies based on electrospinning and wet spinning are widely used in the field of sensors. The fibers and networks, which can be as low as nanoscale, exhibit stable mechanical properties and play a crucial part in the development and manufacturing of flexible electronic devices, there have been several successful cases of fabricating sensors based on Ti_3_C_2_T_*x*_ MXene fibers.^21,76,77^

**Fig. 7:**
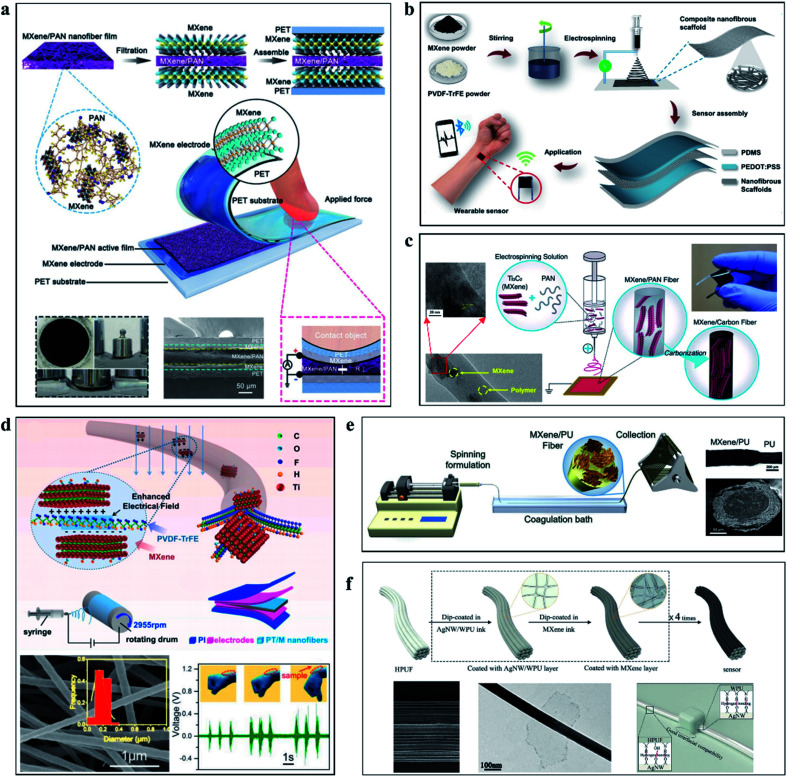
(a), Schematic diagram of a force sensor prepared using an active layer prepared by MXene and PNA nanofibers.^78^ (b) Process diagram of preparing MXene/PVDF TrFF nanofibers for force sensor by electrospinning.^79^ (c) Preparation of MXene/PAN nanofibers by electrospinning for force sensor.^80^ (d) Sensor based on MXene/PVDF TrFF nanofibers and its performance characterization.^81^ (e) Wet spinning MXene/PU multilayer structure fiber.^82^ (f) Schematic diagram of preparation of AgNWs/WPU/MXene fiber.^83^

Electrospinning is a typical manufacturing method of nanofibers, and its simple, high-volume production method can provide a large area of the substrate for Ti_3_C_2_T_*x*_ MXene. Fu *et al.* prepared a 3D polyacrylonitrile (PAN) network by electrospinning and then injected Ti_3_C_2_T_*x*_ MXene into the network by vacuum filtration technology. Based on this, electronic skin with force sensing function was prepared. Fig. 7a shows the preparation process and operation principle of the electronic skin. Using Ti_3_C_2_T_*x*_/PAN network as the active layer, the sensitivity of the device can reach 104 kPa^−1^, the response time (30 ms) and recovery time (20 ms) are also low, and the detection limit is as low as 1.5 Pa.^78^ In the same way, Ti_3_C_2_T_*x*_ MXene and electrospinning solution were mixed in a more direct way to prepare Ti_3_C_2_T_*x*_-containing nanonetworks, which not only did not require the introduction of additives and combination agents but also could create more stable mechanical properties. As shown in Fig. 7b, Sharma *et al.* prepared a nanofiber network containing MXene by electrospinning Ti_3_C_2_T_*x*_ and poly(vinylidene fluoride-trifluoroethylene) (PVDF-TrFE). The sensor has a sensitivity of 0.51 kPa^−1^, a minimum detection limit of 1.5 Pa, and can remain stable for more than 10 000 repeated operations within 0–400 kPa.^79^ Similarly, Fig. 7c and d show the work of Levitt's team and Wang's team, which prepared independent composite networks of Ti_3_C_2_T_*x*_ MXene with PAN and PVDF-TrFE by electrospinning, force sensors based on these networks also demonstrated satisfactory performance.^80,81^

Unlike electrospinning, wet spinning allows more stable components to be added during the production process. The successful wet spinning can obtain controlled single fibers, providing a way to make wearable sensing devices and fabrics. As presented in Fig. 7e, Seyedin *et al.* produced stretchable Ti_3_C_2_T_*x*_ MXene/polyurethane (PU) composite fibers with electrical conductivity and high ductility by wet spinning technology. As a force sensor, its GF can reach 12 900 and the sensing strain can reach 152%.^82^ As presented in Fig. 7f, Pu *et al.* proposed a multilayer fiber sensor composed of silver nanowire (AgNW)/water-based polyurethane (WPU) layer and Ti_3_C_2_T_*x*_ MXene layer by layer self-assembly. With ultra-high sensitivity and a wide operating range, the sensor remains stable over 1000 cycles and has a response and relaxation time of 344 ms.^83^ Existing MXene preparation methods of fiber and network is limited to use wet spinning and electrospinning technology, such as dry spinning,^84,85^ melt spinning,^86,87^ thermal drawing,^88,89^ and other commonly used fiber and network preparation technology have yet to be developed, perhaps this will also lead to the diversification of MXene-based fiber and network applications and the development of high-performance devices such as force sensors for large-area wearable clothing and micro-implantable force sensors. The combination of the fiber manufacturing method and Ti_3_C_2_T_*x*_-based force sensors offers a path to miniaturization, with flexible and sensitive fibers that could be used not only in wearable devices but also in implantable devices and electronic skin. However, the existing research is still limited to large wearable devices. How to combine Ti_3_C_2_T_*x*_ MXene with micro/nano manufacturing and develop a new generation of force-sensing devices by using micromechanical and electrical properties is worth thinking about and exploring.

### 4.3 MXene-based printing inks for force sensors

Printing electronics is expected to become the mainstream manufacturing process of the next generation of flexible electronic products, showing many advantages such as fast, integration, patterning, *etc.* Screen printing, 3D printing, transfer printing, ink writing, inkjet printing and laser patterning in pre-formed Ti_3_C_2_T_*x*_ MXene films are widely used printing technologies. Many forms of printing process have shown their applicability in different applications, but the difficult problem is the preparation and storage of Ti_3_C_2_T_*x*_ MXene-based inks.

MXene in colloidal solution is oxidized more in solution than in powder form, and oxygen dissolved in water is rapidly oxidized to MXene. Excess inert gas (such as nitrogen and argon) is a feasible way to inhibit oxidation, but the water itself, as an oxidant of MXene, will produce a certain oxidation reaction. According to Li *et al.* 's report, storing MXene solution in a vessel filled with argon gas and stored at low temperature and away from light is a feasible long-term storage solution.^90^ In addition, during the printing process, the rheological properties of MXene ink subjected to external stress will significantly affect the printing process availability, repeatability, and overall printing quality.^91^ Adjust the concentration of the solution or change the type of solvent is a feasible solution, but in the face of different printing methods and targets, the required parameters are not the same, it is necessary to timely adjust the strategy to solve the problems in the process of the experiment.

**Fig. 8:**
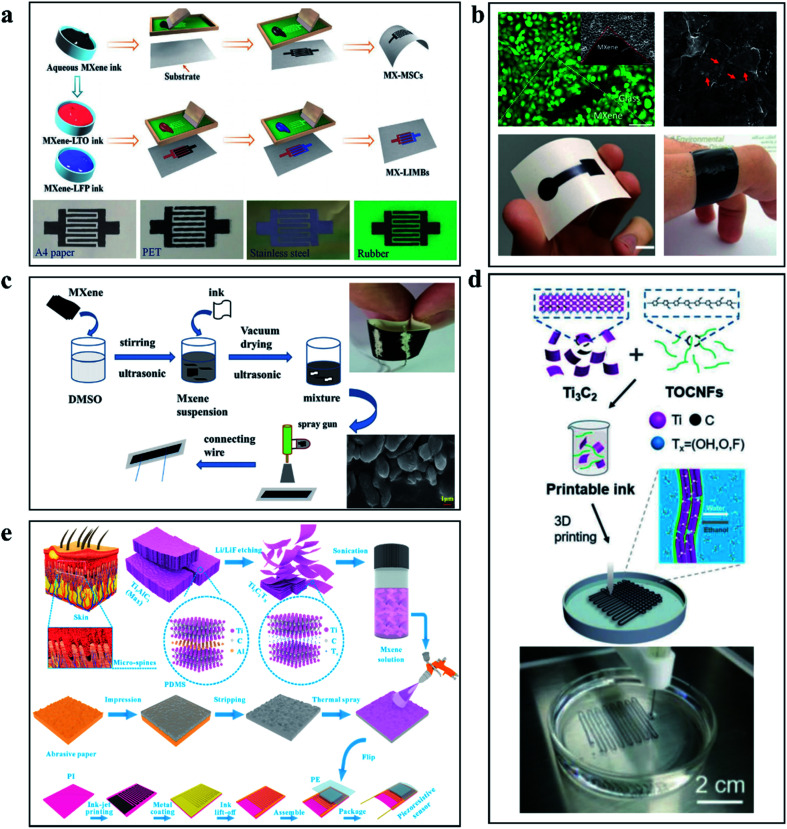
(a) The force sensor circuit prepared by printing with aqueous MXene ink.^92^ (b) Ti_3_C_2_T_*x*_ MXene electrode prepared through the printing process.^93^ (c) The force sensor prepared with MXene ink by spraying techniques.^94^ (d) The force sensor circuit printed with MXene/TOCNFs ink using 3D printing technology.^95^ (e) The bionic structural force sensor prepared by spraying MXene solution.^96^

The use of the printing process has led to a step toward the miniaturization of MXene-based force sensors. As shown in Fig. 8a, Zheng and his colleagues prepared micro-ultracapacitors by forming an additive-free high-capacitance electrode directly from screen printing ink.^92^ Excellent electrical properties (500 S cm^−1^) and force-sensing performance demonstrate excellent performance advantages (areal capacitance ∼1.1 F cm^−2^, record voltage of 60 V, energy density ∼ 154 μW h cm^−2^, response time ∼ 35 ms). Fig. 8b shows the Ti_3_C_2_T_*x*_ MXene electrode prepared by Saleh *et al.* through the printing process, which is uniform and compact with good bending performance.^93^Fig. 8c shows Ma *et al.*'s Ti_3_C_2_T_*x*_ MXene printing process, in which (dimethyl sulfoxide) DMSO was used as a solvent to dissolve Ti_3_C_2_T_*x*_ MXene to produce powder, which was then printed on nylon fabric to form force sensor parts. The GF of the force sensor are 27.2 and 170.9 under the strain of 0–25% and 25–29.7%, respectively.^94^ Rheological properties are a major concern, which will have a significant impact on the printing process and quality of MXene. Cao *et al.* developed hybrid inks of TEMPO (2,2,6,6-tetramethylpiperidine-1-oxylradical)-mediated oxidized cellulose nanofibrils (TOCNFs) and Ti_3_C_2_T_*x*_ MXene inspired by natural materials, the mixed ink has stable rheological properties to achieve accurate structure and fast printing.^95^ Its implementation case is shown in Fig. 8d. Using inkjet printing can be achieved in the thin layer of complex topography printing, this is some strange bionic structure that provides a feasible path. As shown in Fig. 8e, inspired by human skin, Cheng and his team created a highly sensitive piezoresistive sensor. The experimental results demonstrate the development potential of the combination of MXene and inkjet printing technology in the field of biomimetic devices.^96^

Printing technology has shown full potential in the process of force sensor miniaturization and diversification, but there are still many problems to be solved in the printing technology based on Ti_3_C_2_T_*x*_ MXene, such as the diversification of ink storage methods, the mechanism research of fluid rheological properties, the improvement of quality and performance of large-area printing technology, and the diversification of devices. These problems need to be solved in the development of Ti_3_C_2_T_*x*_ MXene printing technology.

### 4.4 MXene based paper and film for force sensors

MXene-based films or papers can be prepared by means of suction filtration. In previous work, MXene-based films and papers have demonstrated considerable folding and tensile properties, which lay the foundation for the preparation of stable and reliable force sensor components.

**Fig. 9:**
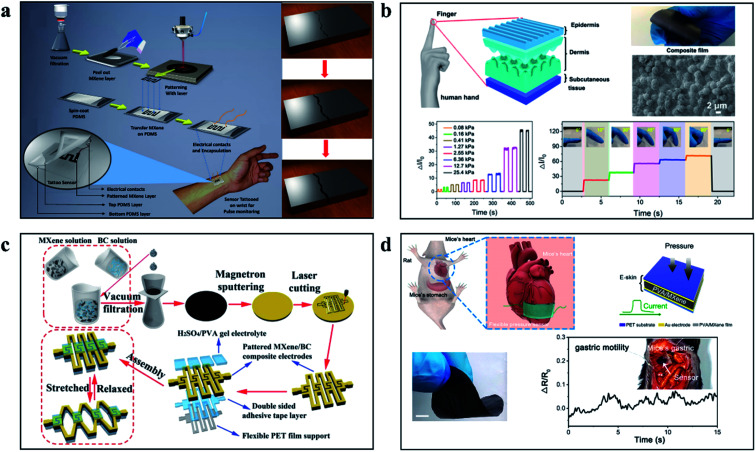
(a) Laser patterned MXene films, using tensile crack change sensing force signals.^57^ (b) Thin film force sensor based on MXene imitating human skin.^54^ (c) A kind of Ti_3_C_2_T_*x*_ MXene/bacterial cellulose (BC) based capacitive force sensors prepared by a composite paper.^97^ (d) Implantable stable high performance force sensor based on MXene film.^98^

By laser patterned Ti_3_C_2_T_*x*_ MXene film, as presented in Fig. 9a, Kedambaimoole *et al.* prepared a shape that was easy to feel the tension and used the strain generated by the force to promote the change of microcracks in the Ti_3_C_2_T_*x*_ MXene device to cause the change of electrical signals to achieve the function of force sensing.^57^ Simple Ti_3_C_2_T_*x*_ MXene film or paper does not meet the requirements of high-performance sensors, biological heuristic sensors have always been a hot topic for researchers to explore, Wang and others develop Ti_3_C_2_T_*x*_/natural microcapsule composite membrane, through the simulation of the human body skin micro/nanoscale structures can be used to make a composite film with the elastic modulus of 0.73 MPa and sensitivity can reach 24.63 kPa^−1^, the response time can be as low as 14 ms and can work under 5000 cycles.^54^Fig. 9b shows the design idea and working principle of the bio-inspired force sensor. In addition, as a potential material for preparing capacitors, film based on MXene has also become an important material. In the work of Jiao and his colleagues shown in Fig. 9c, a kind of Ti_3_C_2_T_*x*_ MXene/bacterial cellulose (BC) has been demonstrated. The capacitive force sensors prepared by a composite paper and an easy to operate laser cutting and patterning technology show good flexibility and sensing performance.^97^ Ti_3_C_2_T_*x*_ MXene used *in vitro* has attracted a lot of attention, but few implantable force sensors based on Ti_3_C_2_T_*x*_ MXene have been reported. The reasons are as follows: firstly, the hydrophilicity and oxidation of Ti_3_C_2_T_*x*_ MXene are unstable in the human body; secondly, the volume of existing sensors is too large to be implanted. In the work shown in Fig. 9d, Zhao *et al.* reduced the diffusion coefficient of Ti_3_C_2_T_*x*_ MXene in acidic and alkaline solutions by introducing PVA, increased the adsorption (free) energy, and provided a mixed film with stable chemical stability. After 8 weeks of the constant pressure of 0.85 kPa, the sensitivity decreased by 8.5%. In water and pH = 3 and pH = 11 aqueous solutions, the performance decreased by 17.7%, 32.6% and 34.7%, respectively. The biocompatibility and practical force signal sensing performance of the sensor was verified by implanting the sensor into mice.^98^

### 4.5 Other works

The special properties of MXene allow it to be used in force sensors in a variety of forms. In addition to aerogels and hydrogels, fibers and networks, inks, paper, and films, some work has been focused on exploring the diverse development of MXene.

**Fig. 10:**
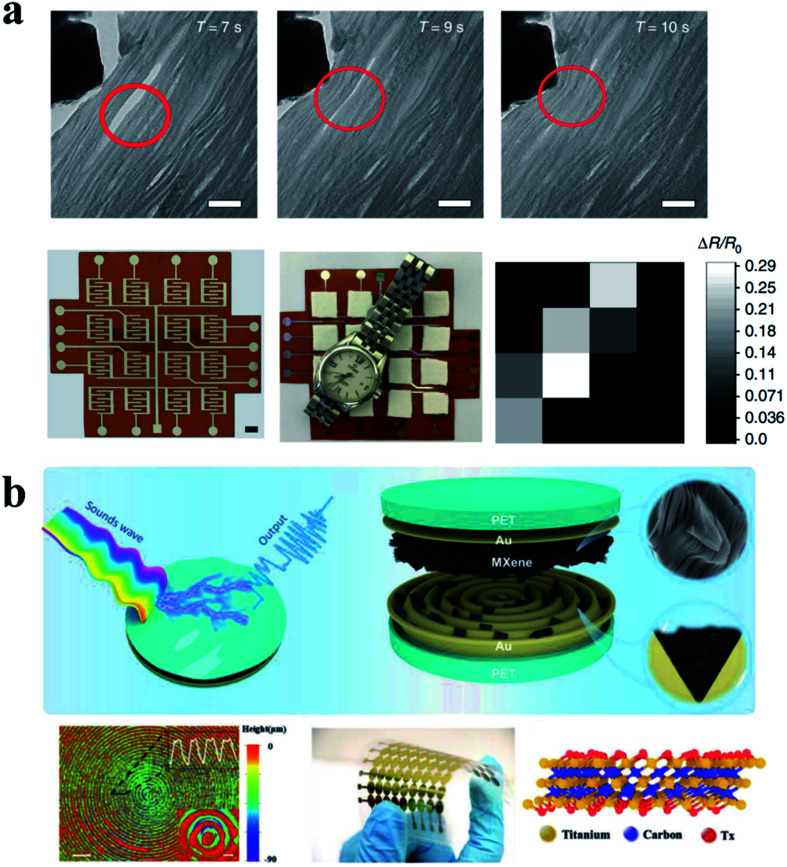
(a) Principle and device diagram of force sensor based on MXENE layer spacing variation.^53^ (b) Structure diagram of MXENE flexible sensor based on fingerprint dimension structure.^99^

Materials with piezoresistive properties developed with MXene have been widely reported. However, no one has discovered or used the basic characteristics. The interlayer distances of MXene can vary significantly under external pressure, resulting in changes in electrical resistance. Based on this basic characteristic, Ma *et al.* reported a piezoresistive sensor with high flexibility and sensitivity, which showed high sensitivity (∼180.1), fast response (<30 ms), and reversible compression.^53^ The principle and working diagram of the equipment are shown in Fig. 10a. Gao and colleagues have developed a new flexible piezoresistive sensor based on microchannel constraints that enable simultaneous pressure, sound, and acceleration of multiple micro-force sensors. The sensor benefits from the synergistic effect of the fingerprint microstructural channel and the accordion microstructural MXene material to achieve a detection limit of 9 Pa, high sensitivity of 99.5 kPa^−1^, a fast response time of 4 ms, and unattenuated durability of over 10 000 cycles.^99^ The structure and working diagram are shown in Fig. 10b. It is worth noting that the combination of MXene with a highly innovative load-bearing structure enables the development of high-performance force sensors with a variety of practical applications, which will greatly enhance the diversity and possibilities of MXene development and application. To more visually demonstrate the performance of the MXene-based force sensors, the relevant performance data are shown in Table 2.

**Table 2:** The performance data of the force sensors based on MXene

	Type	Working rage	Sensitivity (kPa^−1^)	GF	Response time (ms)	detection limit	cyclic stability	Ref.
Hydrogel (Ti_3_C_2_T_*x*_/PVA)	Capacitive	∼1200% (tensile)	∼0.40	—	—	—	10 000	67
Hydrogel (Ti_3_C_2_T_*x*_)	Tensile	∼1810% (tensile)	—	—	380	—	500	68
Hydrogel (Ti_3_C_2_T_*x*_)	Tensile	∼3400% (tensile)	—	80	—	—	—	56
Hydrogel (graphene/Ti_3_C_2_T_*x*_)	Tensile	2000% (tensile)	—	2.4	—	—	—	69
Aerogel (Ti_3_C_2_T_*x*_/ANF)	Piezoresistive	2.0–80.0% (compressive)	128	—		100	—	72
AgNWs/Ti_3_C_2_T_*x*_	Piezoresistive	—	645.69	—	—	1.25	10 000	74
Aerogel (Ti_3_C_2_T_*x*_/RGO)	Piezoresistive	—	22.56	—	<200	10	10 000	55
MXene nanocomposite organohydrogel	Piezoresistive	1000% (tensile)	—	44.85	—	—	—	60
Ti_3_C_2_T_*x*_/PAN	Piezoresistive	—	104	—	30	1.5	10 000	78
Ti_3_C_2_T_*x*_/PVDF-TrFE	Piezoresistive	<400 kPa (pressure range)	0.51	—	—	1.5	10 000	79
Ti_3_C_2_T_*x*_/PU	Tensile	152% (tensile)	—	>100	344	—	1000	83
Ti_3_C_2_T_*x*_/DMSO ink	Tensile	—	—	27.2/1170.9	—	—	2950	94
Ti_3_C_2_T_*x*_	Piezoresistive	—	151.4	—	<130	4.4	10 000	96
Ti_3_C_2_T_*x*_/natural microcapsule	Piezoresistive	730 kPa (pressure range)	24.63	—	14	14	5000	54
Ti_3_C_2_T_*x*_	Piezoresistive	—	—	180.1	<30	—	4000	53
Ti_3_C_2_T_*x*_	Piezoresistive	—	99.5	—	4	9	10 000	99

## 5. Conclusion and prospect

In conclusion, many Ti_3_C_2_T_*x*_ MXene-based force sensors are reported as mentioned in this review. From the perspective of hydrogel and aerogel, fiber and network, ink and printing, film and paper, we have enumerated important cases and introduced their preparation strategies, properties, and applications.

Although Ti_3_C_2_T_*x*_ MXene-based force sensors have shown favorable performance and potential, there are still many problems to be explored and solved. First of all, the oxidation of MXene is still a difficult problem to solve, the sensor will inevitably be oxidizable during the working, which will greatly reduce the stability and service life of the device. The oxidation resistance of Ti_3_C_2_T_*x*_ MXene based sensors is a question worthy of further investigation. Secondly, the design of Ti_3_C_2_T_*x*_ MXene force sensors is still limited to the resistance and capacitance changes caused by force compression and tension. How to create a large-scale spatial force distribution perception and force memory integrated thin-film force sensing system needs to be tried and explored. Third, the diversity of Ti_3_C_2_T_*x*_ MXene features makes it can exist in various forms, such as gels, fibers, networks, and thin films. Although many achievements have been made, the lack of forms and modes highlights the diversity, flexibility, and transparency of force sensors. The development of flexible and transparent force sensors based on Ti_3_C_2_T_*x*_ MXene may be a worthy approach. Some works on transparent electrodes based on MXene have been reported. This also provides some feasible reference cases for how to find a unique path to combine MXene with other materials to design multi-function sensors with flexibility, transparency, and other special functions based on Ti_3_C_2_T_*x*_ MXene. The existence of piezoelectric properties of MXene materials is reported.^100^ It is worth further research whether it can further explore the piezoelectric properties of MXene materials and develop a series of piezoelectric sensors based on MXene. The repeatability of the dynamic response of MXenes and its significant piezoresistive behavior have been proposed and investigated.^101^ Based on this phenomenon, the development of dynamic sensors for measuring pressure change, acoustic emission, structural vibration and other dynamic phenomena is also worth exploring.

As an emerging two-dimensional material with great potential, Ti_3_C_2_T_*x*_ MXene has great potential in the application of force sensors. The variety of morphology, good electrical conductivity, and excellent mechanical properties make it a special place in a wide variety of 2D materials family. A solid foundation has been laid for the existing work although many problems remain to be solved.

## Conflicts of interest

There are no conflicts to declare.

## Supplementary Material
